# Immediate placement and provisionalization of an implant after removal of an impacted maxillary canine: two case reports

**DOI:** 10.1186/s40729-015-0013-3

**Published:** 2015-05-30

**Authors:** Elise G Zuiderveld, Henny J A Meijer, Arjan Vissink, Gerry M Raghoebar

**Affiliations:** 1 Department of Oral and Maxillofacial Surgery, University of Groningen, University Medical Center Groningen, P.O. Box 30.001, , NL-9700 RB Groningen, the Netherlands; 2 Department of Fixed and Removable Prosthodontics, University of Groningen, University Medical Center Groningen, P.O. Box 30.001, , 9700RB, Groningen, the Netherlands

**Keywords:** Dental implants, Maxillary impacted canine, Immediate implant, Immediate placement, Immediate provisionalization

## Abstract

Single immediate implant replacement is accompanied by excellent survival rates and a favorable esthetic outcome. The objective of this report was to describe a surgical approach for removal of a buccal or palatally located impacted secondary canine, combined with extraction of the failing primary canine, and immediate placement and provisionalization of an implant. A window technique was applied for surgical removal of the impacted canine. The alveolar crest was preserved. After extraction of the primary canine, the implant was inserted with primary stability. Finally, the exposed surfaces of the implant were covered with a 1:1 mixture of autologous bone and Bio-Oss®. At the 1-year evaluation, both implants were successfully osseointegrated and in function. Esthetics were excellent. It is concluded that under premise of preservation of sufficient bone to achieve primary stability of the implant, removal of the canines can be combined with immediate placement and provisionalization of the implant.

## Background

Maxillary canines are the second most impacted teeth (20 % of all impacted teeth); the prevalence in general population is approximately 2 %. Most impacted cuspids are located palatally, with a palatal/buccal ratio of 8:1 [1–4].

There are several known treatment options for impacted canines to align them into the dental arch. The most widely used option is orthodontic traction after surgical exposure. An alternative is autotransplantation of the impacted canine optionally combined with orthodontic treatment, e.g., when only orthodontic repositioning is not possible or unsuccessful [4–6]. In general, these treatment options use the patient’s own teeth to encounter the clinical problem. The advantages of this aspect are functioning as normal teeth, normal dentofacial development, and maintenance of the alveolar bone. Prognosis of autotransplantation is significantly dependent on the stage of root development, with lower risk of failure in teeth with open apex [7]. Success rates for autotransplantation, mentioned in the literature, lie between 82 and 99 % [8]. Disadvantages of both treatment options for alignment of impacted canines into the dental arch are a long treatment time and high costs, not to mention the unpredictable final outcome [2, 9, 10]. Surgical exposure followed by orthodontic traction is associated with damage to supporting structures such as bone loss, root resorption, and gingival recession [2, 10]. The most frequently reported complications in autotransplantation are root resorption or ankylosis, pulp necrosis, and reduction of final root length [5, 7]. Replacement of a failing single tooth, such as a failing primary canine with an impacted secondary canine, through a single implant is another reasonable treatment option [3, 4, 6]. Single implant treatment in this respect is not widely applied yet, but should be considered, if orthodontic treatment and autotransplantation are not feasible because of factors such as canine location, severity of impaction and age of the patient, or when the patient is not willing to encounter conventional treatment options because of treatment duration, morbidity, and costs [4, 6, 9]. With regard to prosthodontic rehabilitation of a single implant, the concept of immediate single implant placement and provisionalization is not yet a standard treatment [11], but there is a growing interest in immediate tooth replacement, particularly in the esthetic region.

Applying an immediate protocol means shortening of the treatment duration as only one surgical intervention is needed and no need for a temporary prosthesis. Thus morbidity and costs of the treatment are reduced [12–14].

According to the literature, immediate implant placement is accompanied by survival rates comparable to conventionally placed implants [12, 15]. With regard to immediate provisionalization, it is not yet set that the esthetic outcome is more favorable [12]. However, immediate provisionalization of the immediately placed implant is presumed to give better support to the surrounding peri-implant tissue for preservation of the original architecture, conditions which are in favor for an optimal esthetic result [3, 11, 15, 16].

The objective of the present report was to describe a surgical approach for removal of an impacted secondary canine, either located buccally or palatally, combined with extraction of the primary canine and immediate placement and provisionalization of an implant.

## Case presentation

### Case 1

**Fig. 1:**
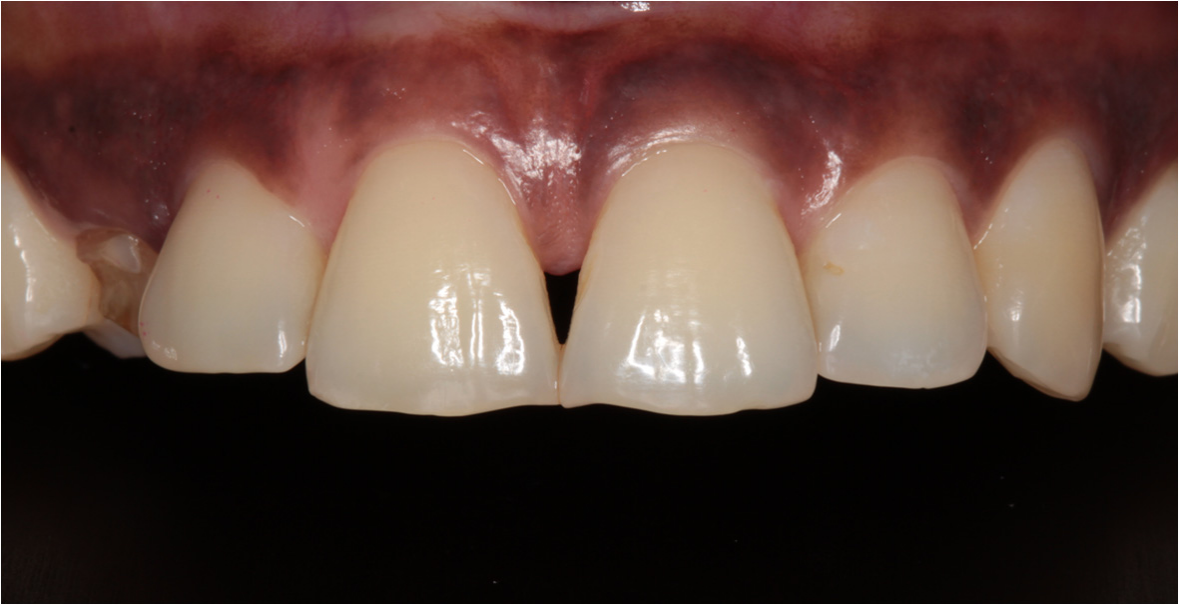
Clinical view showing the failing right primary canine

A 36-year-old woman consulted the Department of Oral and Maxillofacial Surgery of the University Medical Center Groningen, Groningen, the Netherlands, with a persisting upper right primary canine and impacted secondary canine (Fig. 1). The primary canine had to be removed because of fracture of the crown. The patient did not want to undergo orthodontic treatment, and autotransplantation was no treatment option. She asked for a fixed restoration without involvement of the adjacent teeth and chose for a single implant treatment. She was healthy and non-smoker.

Intra-oral examination revealed a healthy, well maintained dentition. Clinically, adequate bone volume was thought to be present at the future implant site as well as favorable conditions for an implant crown with an anatomical design.

**Fig. 2:**
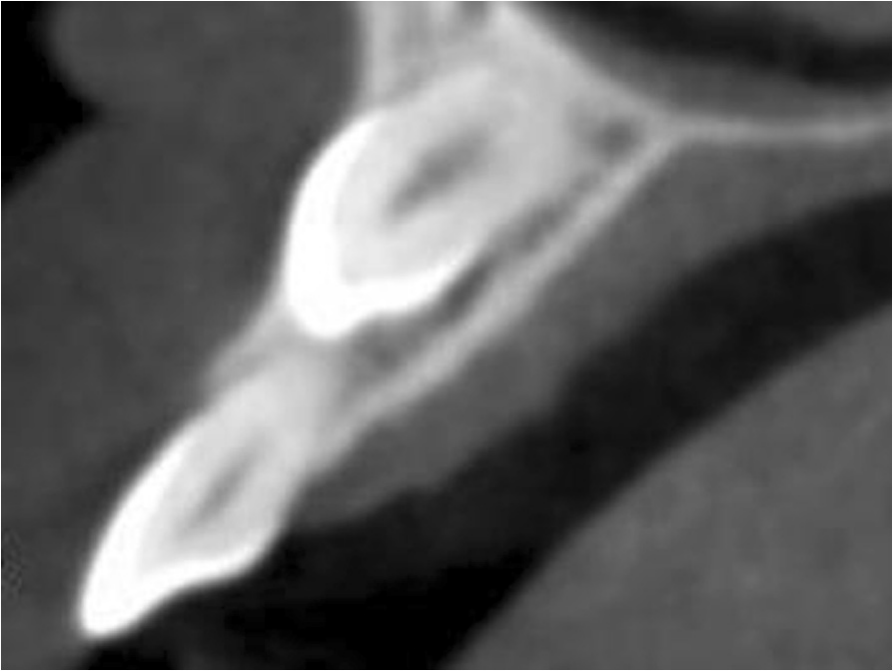
CBCT image showing the buccal location of the impacted secondary canine

Radiographic examination, consisting of a standardized digital intra-oral radiograph and a cone beam computer tomography (CBCT) image (i-CAT® 17–19; Imaging Sciences International, LLC, Hatfield, USA), was done prior to localize the impacted canine. The CBCT image revealed an impacted right maxillary canine, situated on the buccal side (Fig. 2) with sufficient bone volume on the apical part of the future implant site. Removal of the impacted canine seemed to be possible with maintenance of sufficient bone at the future implant site for immediate implant placement. Furthermore, no pathology of the dentition was pre-existent. Because of this favorable starting point, it was decided to extract the primary canine and to surgically remove the secondary canine, immediately followed by insertion of an implant according to an immediate loading protocol.

Preoperatively, a cast was made for planning the preferred position of the implant from a prosthodontic perspective. Next, a transparent acrylic resin template (Vertex Castapress; Vertex-Dental BV, Zeist, the Netherlands) was made of this cast with the future implant crown in the preferred position. This template was transferred to a surgical guide. Care was taken to design the surgical guide as such that the guide channel allowed for screw retaining of the provisional restoration.

**Fig. 3:**
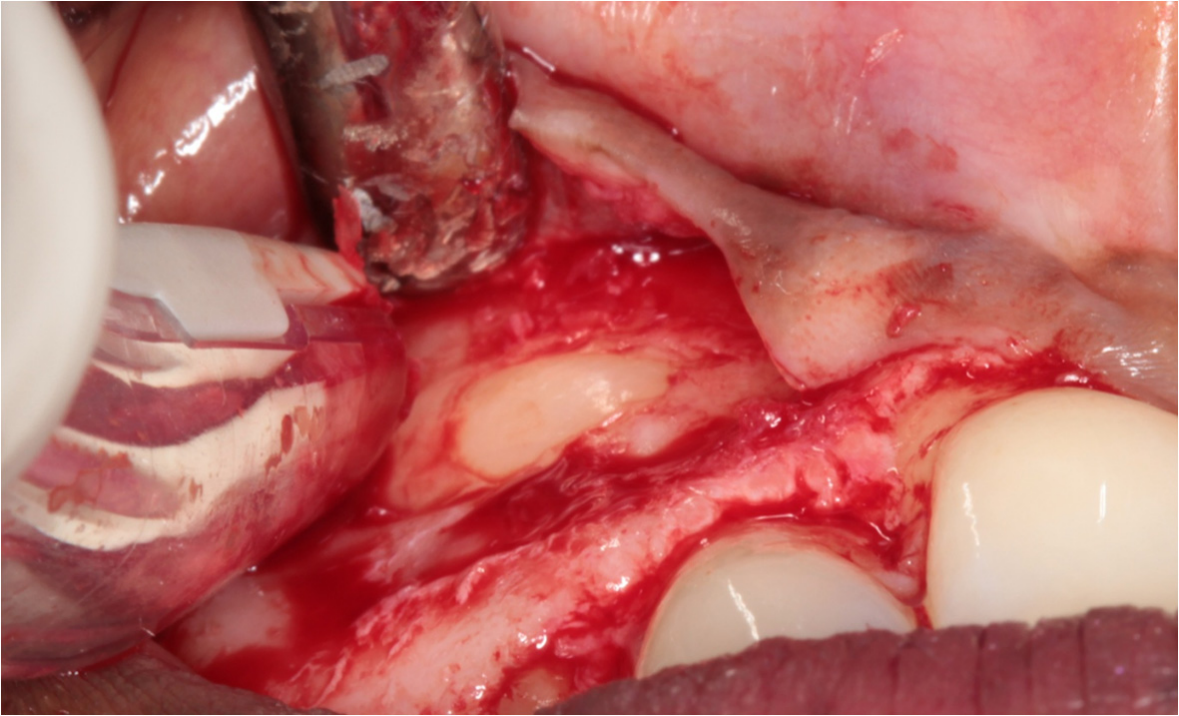
The impacted canine has become visible after elevation of a full-thickness buccal mucoperiosteal flap and removing overlying bone

One day before surgery, the patient started taking antibiotics (amoxicillin 500 mg, three times daily for 7 days) and using a 0.2 % chlorhexidine mouthwash (Corsodyl; GlaxoSmithKline, Utrecht, the Netherlands) for oral disinfection. Following the administering of local anesthesia (Ultracaine D-S Forte; Aventis Pharma Deutschland GmbH, Frankfurt am Main, Germany), an incision was made on the palatal side of the crest with extensions in the buccal and palatal sulcus of the adjacent teeth. For good access to the impacted maxillary canine, a full-thickness buccal mucoperiosteal flap was elevated. After removal of bone overlying the impacted canine with a round drill and a disposable bone scraper (Safescraper® TWIST Cortical Bone Collector, Biomet 3i, Palm Beach Gardens, USA), the impacted tooth (Fig. 3) was extracted with preservation of the alveolar crest. The roots of the adjacent teeth were not exposed. Finally, the primary canine was extracted with a forceps in order to preserve the alveolar crest as long as possible.

**Fig. 4:**
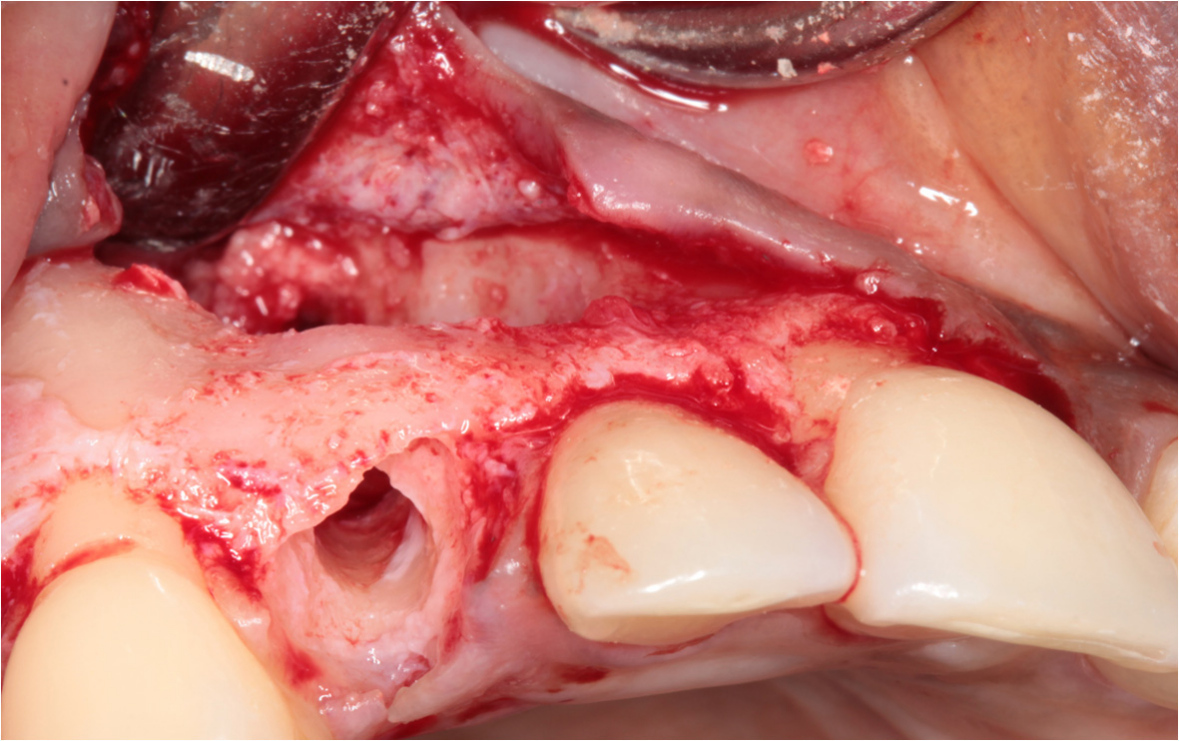
The prepared implant socket and osseous defect resulting from removal of the buccally impacted secondary canine and the primary canine. Note that the upper part of the alveolar crest is intact

**Fig. 5:**
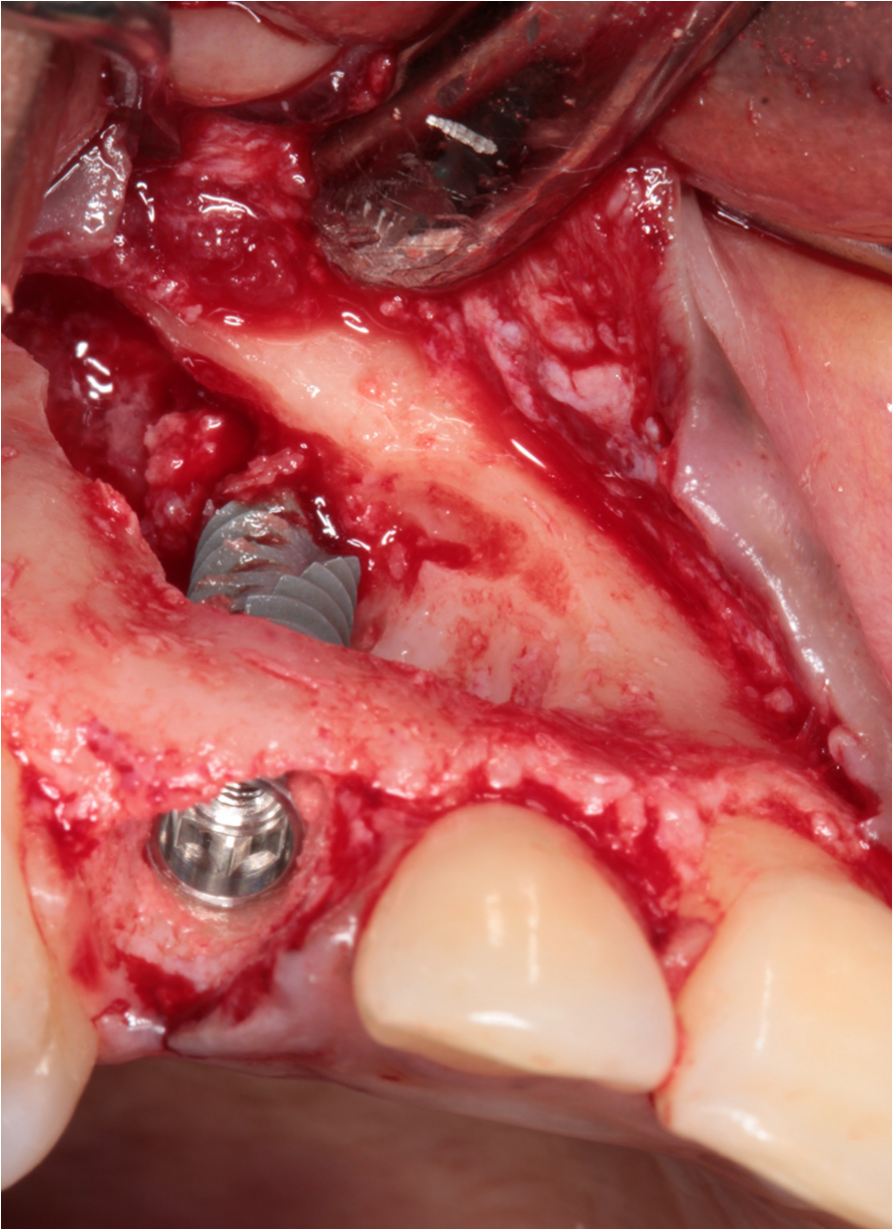
The implant is placed in the prepared socket

Sufficient bone was left on the buccal and palatal side of the alveolus to allow for immediate implant placement (Fig. 4). After drilling the implant site according to the implant system applied, the final twist drill was placed in the prepared socket. Next, the space between the twist drill and the palatal bone wall was augmented with a 1:1 mixture of autologous bone, harvested from the retro molar area, and Bio-Oss® (Geistlich Pharma AG, Wolhusen, Switzerland). Next, the twist drill was carefully removed and an implant (NobelActive RP, 18 mm; Nobel Biocare AB, Göteborg, Sweden) was placed into the prepared implant socket according to the procedure prescribed by the manufacturer, guided by the surgical template (Fig. 5). An 18-mm implant was chosen for good primary stability because of the bone defect. The shoulder of the implant was placed at a depth of 3 mm apical to the buccal and cervical aspect of the prospective clinical crown to provide soft tissue to develop an adequate emergence profile. Good primary implant stability of >45 Ncm was obtained, determined with the measuring device for implant site preparation (Osseocare; Nobel Biocare AB).

**Fig 6:**
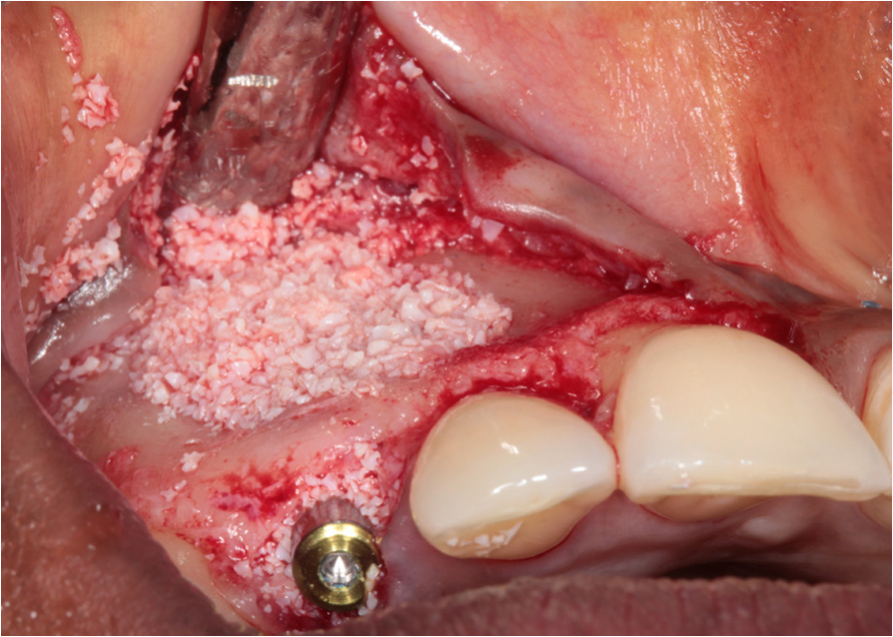
Situation after implant placement and restoration of the bony defect with a 1:1 mixture of Bio-Oss® and autologous bone

Next, an open tray impression was made at implant level using a custom acrylic resin impression tray (Lightplast base plates; Dreve Dentamid GmbH, Unna, Germany) and a polyether impression material (Impregum Penta; 3 M ESPE, St. Paul, USA). Finally, a healing abutment (NobelReplace; Nobel Biocare AB) was placed, and any remaining residual space between the implant and the buccal bone wall was filled with a 1:1 mixture of autologous bone and Bio-Oss® (Geistlich Pharma AG) (Fig. 6). A Geistlich Bio-Gide (Geistlich Pharma AG) was used to cover the reconstructed alveolar process. The wound was closed with Ethilon 5–0 nylon sutures (Johnson & Johnson Gateway, Piscatatway, USA).

**Fig. 7:**
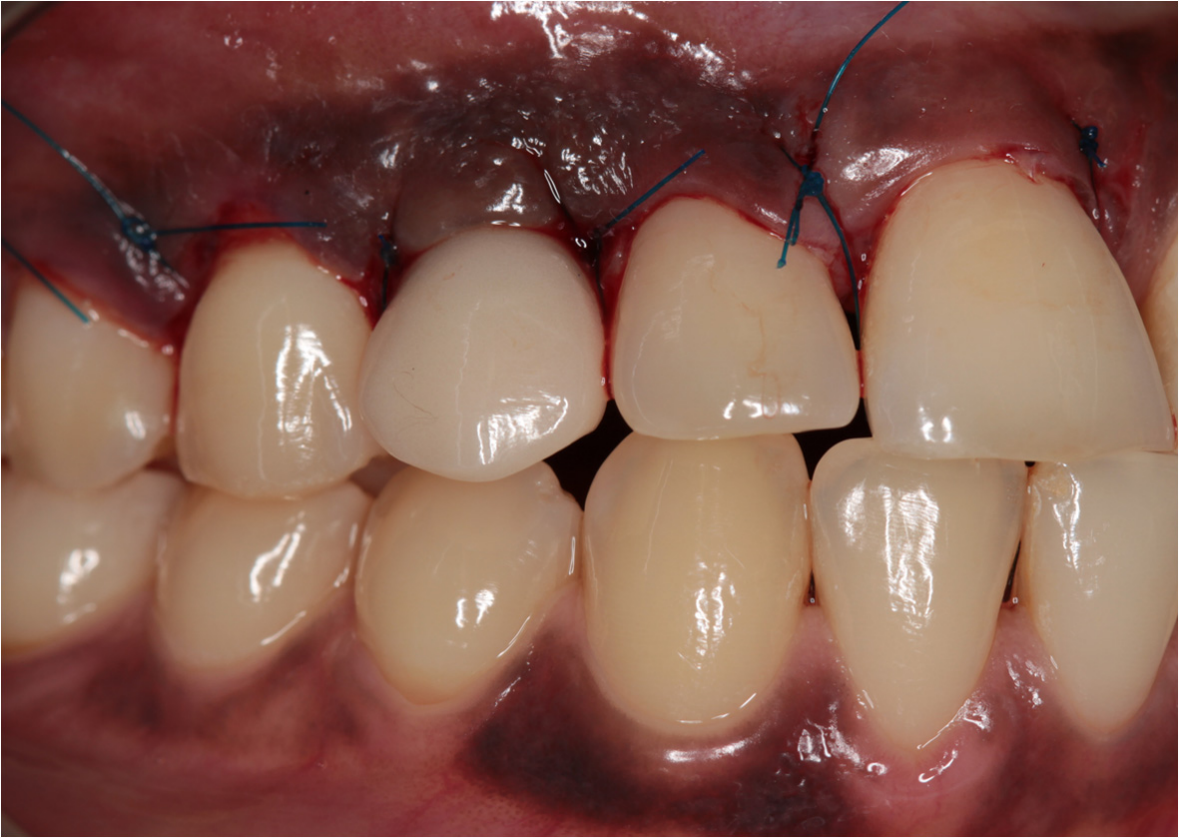
Clinical view immediately after placement of the provisional implant crown

Six hours following implant placement, the healing abutment was removed, and a provisional crown was placed and torqued to 32 Ncm (Fig. 7). Special care was taken to prevent any contact with the antagonist teeth as well as that the provisional restoration was contoured for optimal support of the peri-implant soft tissue. In particular, the interproximal papillae were given sufficient space to regenerate.

The patient was instructed to follow a soft diet, to avoid exerting force on the provisional restoration, and to continue the chlorhexidine rinse (Corsodyl; GlaxoSmithKline) for 7 days. For pain control, 600 mg ibuprofen (Brufen Bruis 600; Abott BV, Hoofddorp, the Netherlands) was prescribed, to be taken three times daily for the time period needed. Two weeks following surgery, the sutures were removed.

**Fig. 8:**
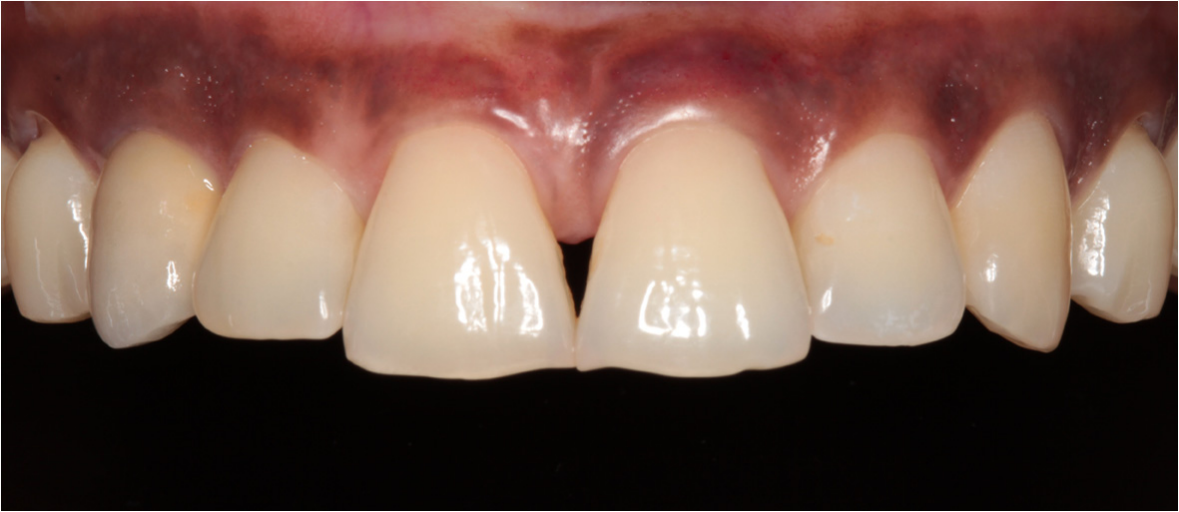
Clinical view showing optimal esthetics around the screw-retained definitive all-ceramic crown

**Fig. 9:**
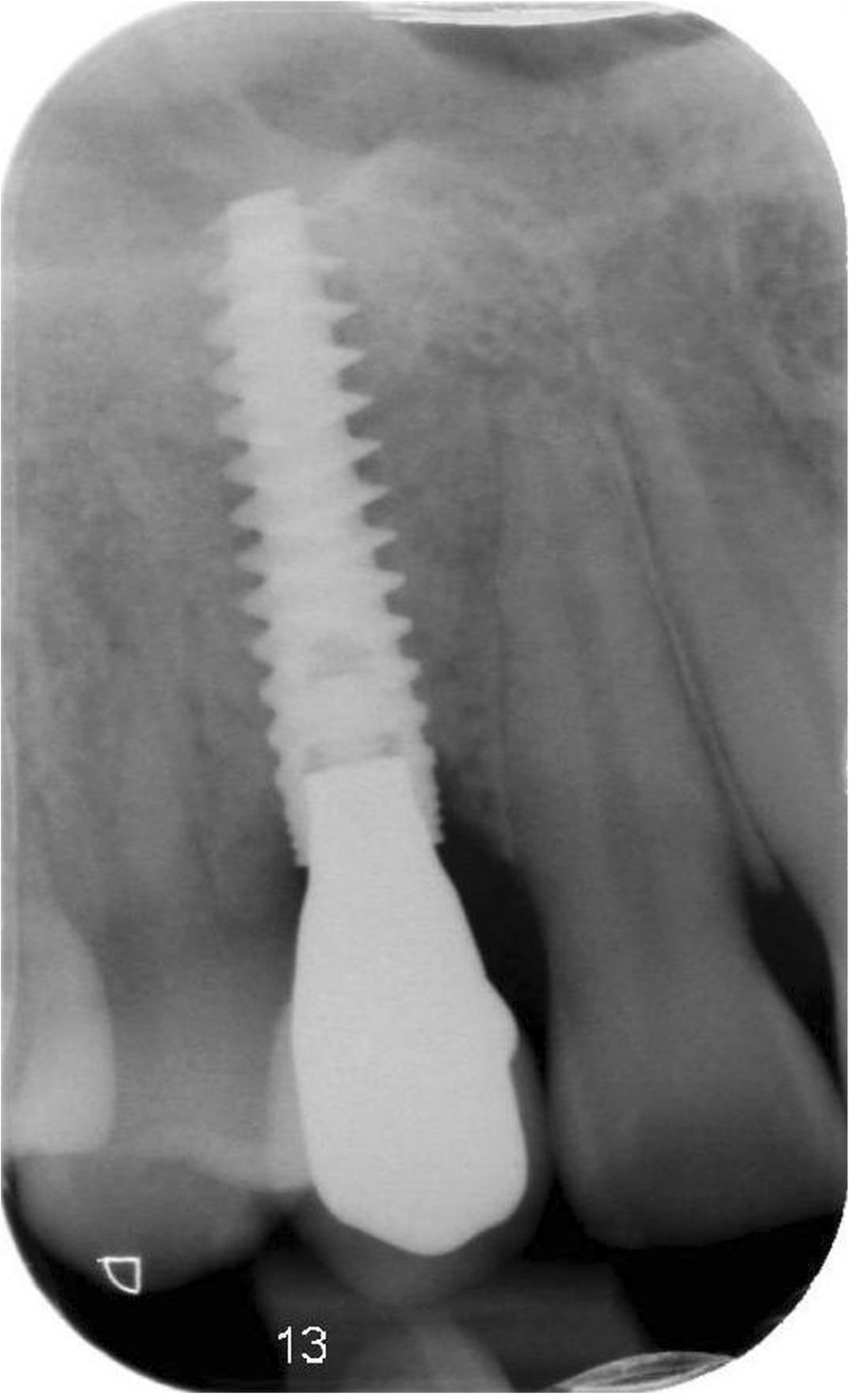
Intra-oral radiograph showing the implant 12 months after placement

Three months later, a screw-retained definitive all-ceramic crown was placed. Follow-up appointments were scheduled 1 and 12 months after installation of the definitive implant crown (Fig. 8) and consisted of intra-oral examination and radiographic assessment of the peri-implant bone level. At both follow-up visits, intra-oral examination revealed healthy peri-implant tissues. Radiographic examination showed minimal bone resorption mesial and distal of the implant (Fig. 9).

### Case 2

**Fig. 10:**
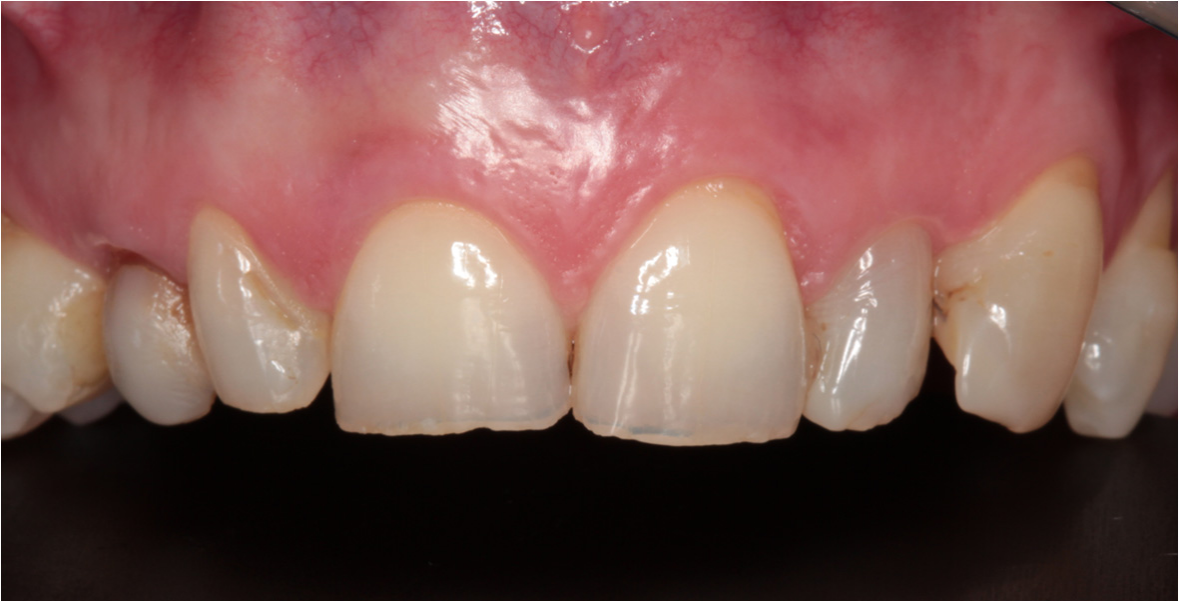
Clinical view showing the failing right primary canine

**Fig. 11:**
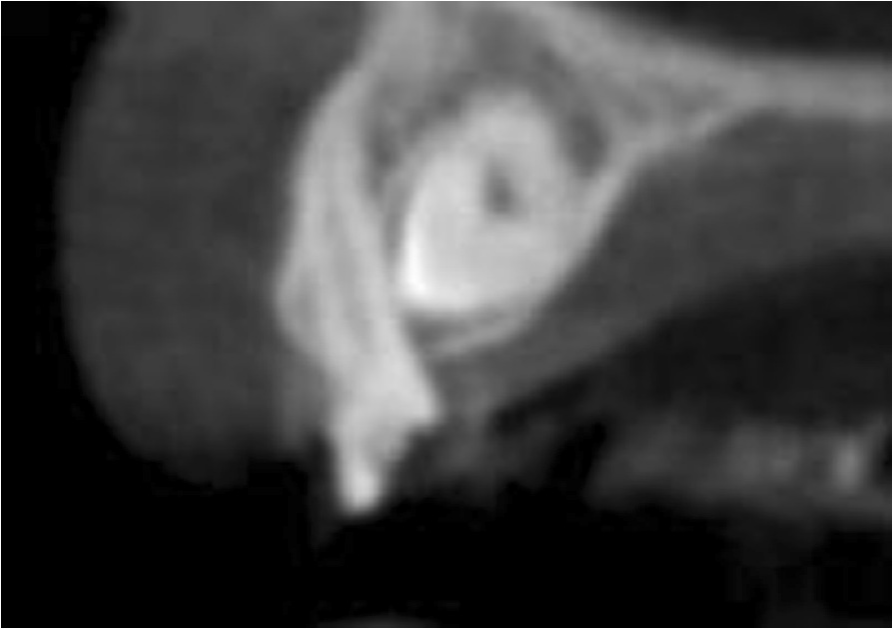
CBCT image showing the palatal location of the impacted secondary canine

A 45-year-old man consulted our department with an impacted right maxillary canine and a persistent primary canine with evident mobility and in need of removal (Fig. 10). The patient chose for a single implant treatment because he wanted to have a long lasting and fixed solution for the failing tooth. All general health prerequisites were met and intra-oral examination revealed a healthy, well-maintained dentition. Clinically, adequate bone volume was present at the future implant site. In all dimensions, sufficient space was available for an implant crown with an anatomical design. The CBCT image (i-CAT® 17–19) revealed an impacted canine situated on the palatal side (Fig. 11) without any other pathology of the dentition as well as sufficient bone volume on the apical part of the future implant site.

**Fig. 12:**
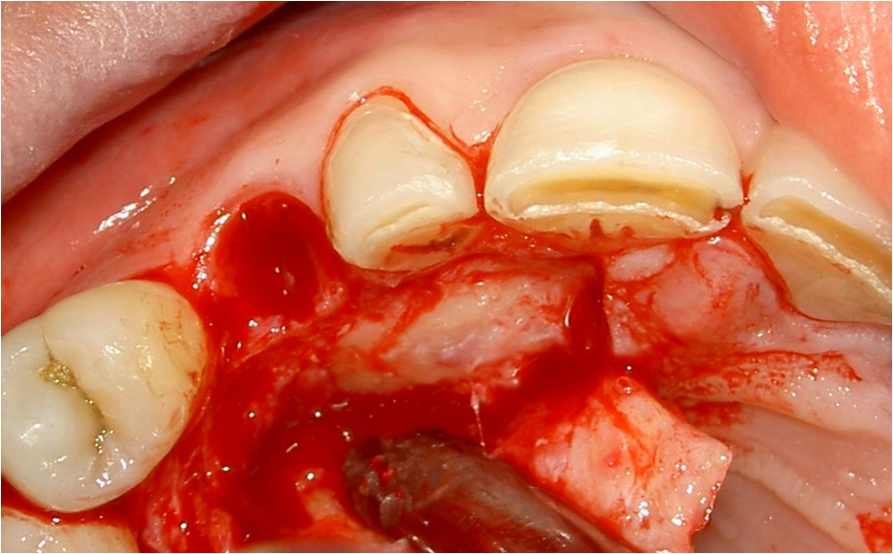
The impacted canine has become visible after elevation of a full-thickness palatal flap and removing overlying bon

Preoperative preparations were the same as for the first case. Next, after administration of local anesthesia (Ultracaine D-S Forte), a full-thickness palatal flap, by an intrasulcular incision on the palatal gingiva from the distal margin of the first premolar to the mesial margin of the central incisor, was elevated for good access to the impacted canine (Fig. 12). Extraction was done carefully, again using a round drill and a bone scraper (Safescraper®), with preservation of the alveolar crest and buccal bone wall. The roots of the neighboring teeth were not exposed. Afterwards, the primary canine was extracted using a forceps.

**Fig. 13:**
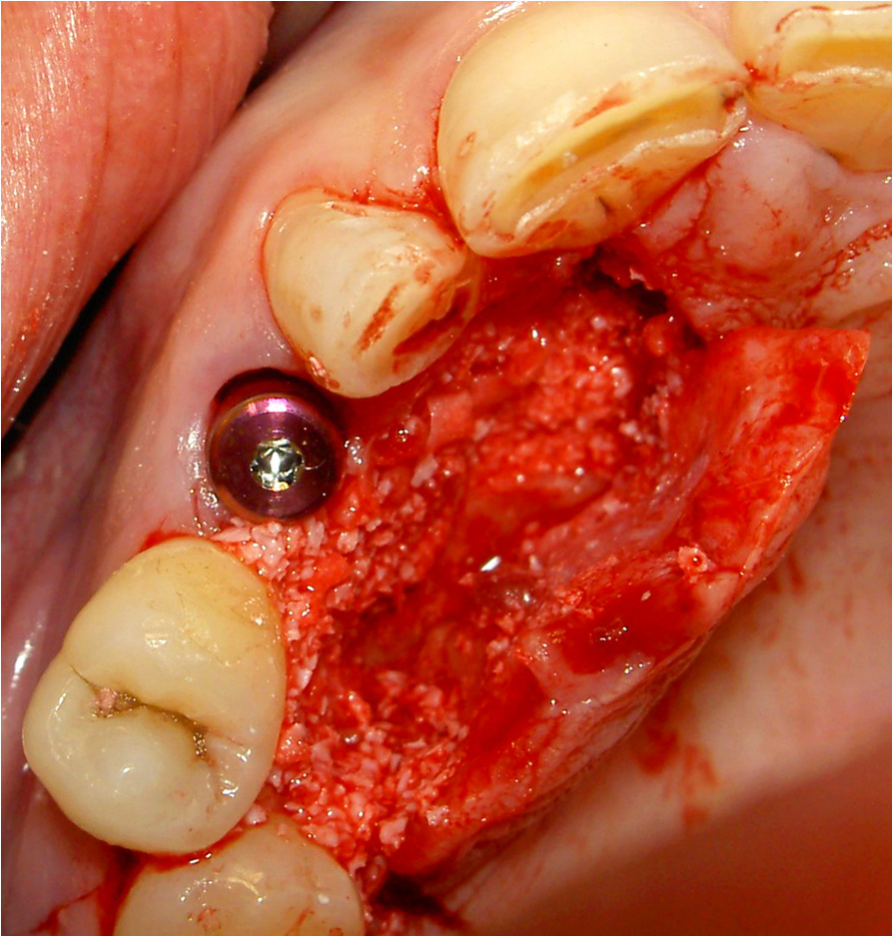
Situation after implant placement and repair of the bony defect with a 1:1 mixture of Bio-Oss® and autologous bone

Because of sufficient bone remaining, an implant (NobelActive NP, 18 mm) was placed immediately into the extraction socket according to the prescribed manufacturer’s procedure and guided by the surgical template, with good primary stability of >45 Ncm. An 18-mm implant was chosen for good primary stability because of the bone defect. Bone augmentation was done as described in the first case (Fig. 13). Installation of the provisional implant crown, about six hours following implant placement, was also done according to the procedure described for the first case and with special attention to avoid any contact to the antagonist and contour of the crown. Post-operative care instruction was identical to the first case too. Sutures were removed two weeks after implant placement. Three months after implant installation, the definitive implant crown was placed.

**Fig. 14:**
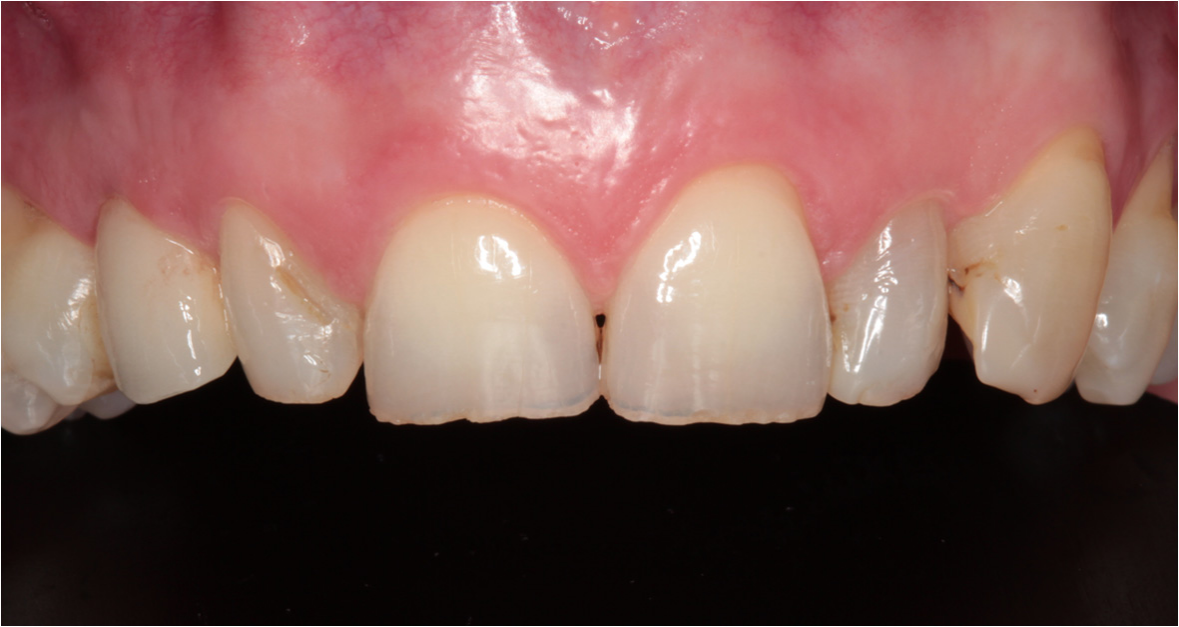
Clinical view showing optimal esthetics around the screw-retained definitive all-ceramic crown

**Fig. 15:**
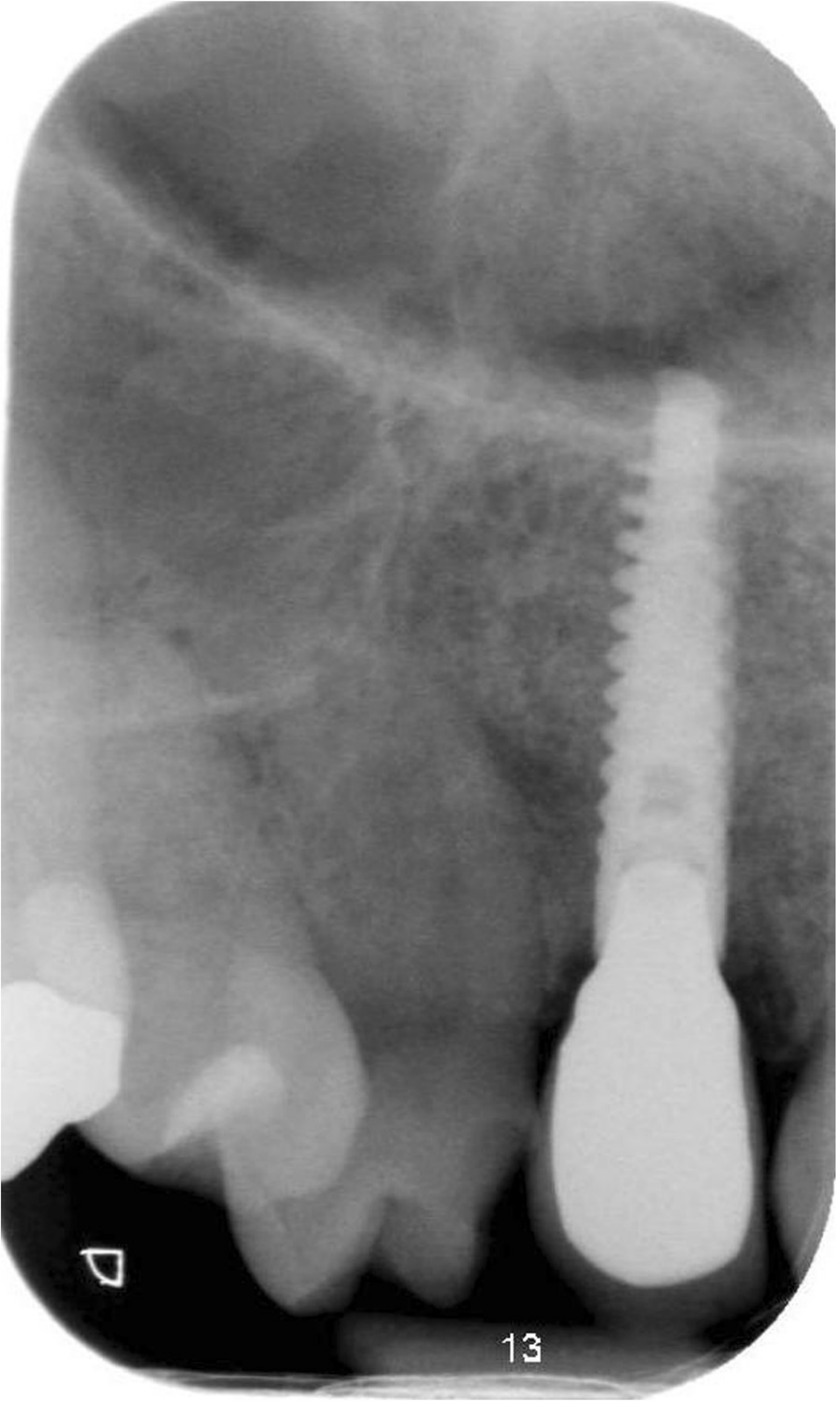
Intra-oral radiograph showing the implant 12 months after placement

During both follow-up appointments, scheduled 1 and 12 months after installation of the final implant crown (Fig. 14), intra-oral examination revealed healthy peri-implant tissues. Radiographic examination showed minimal bone resorption mesial and distal of the implant (Fig. 15).

### Discussion

This case report describes two approaches for immediate replacement of a failing primary canine and an impacted secondary canine, viz. one for impacted cuspids located at the buccal side of the maxilla and one for impacted cuspids located at the palatal side of the maxilla, by an immediately placed and provisionalized single implant. With both approaches, esthetically satisfying results were achieved, comparable with other case reports [3, 6, 10]. Removal of an impacted canine causes an evident bone defect, which can be a possible limitation of the proposed technique, because it may be difficult or even not possible to place the implant with enough primary stability [6].

In order to preserve as much bone as possible during the surgical removal of the impacted tooth, it is important to localize the impacted tooth and to judge whether a buccal or palatal approach will preserve most of the native bone by three-dimensional radiographical imaging. Such an approach is supported by recent literature claiming that evaluation of a CBCT image favors treatment planning [5, 9]. Even though in both cases, a significant amount of bone had to be removed to expose the impacted cuspid, proper pre-operative planning still allowed for immediate implant placement and immediate provisionalization because of enough primary stability of the implant.

Characteristic for the bone defect in both cases was preservation of the coronal part of the alveolar crest, especially the labial bone plate. According to Kan et al. the presence of ideal pre-existent soft and hard conditions are a prerequisite for immediate implant placement and provisionalization. Particularly, an intact labial bone plate is important to minimize facial gingival recession [17, 18].

Furthermore, an implant system was used, which claims to achieve good primary stability in a small amount of bone, which allowed for immediate provisionalization [19, 20]. This is consistent with recent literature that claims when good primary implant stability is achieved, in the presence of sufficient bone volume, single implants should be provisionalized immediately for preservation of the pre-operatively existing tissue conditions in order to achieve a favorable esthetic outcome [12, 21–24].

But, in order to avoid significant facial hard and soft tissue loss due to the remodeling process after tooth extraction, jeopardizing the final esthetic result, a bone grafting procedure is necessary [17].

Although immediate implant placement and provisionalization is a desired treatment option, it is not possible to apply to young still growing patients. In this case, orthodontic treatment or autotransplantion are indicated [8].

In addition, a primary canine is smaller in all dimensions, but especially in its mesio-distal dimension, compared to a secondary canine. In order to place an implant crown with comparable dimensions as the contralateral secondary canine to achieve symmetry, sufficient mesio-distal space is needed. This can be a limitation of the proposed technique because when insufficient space is available, orthodontic treatment is still needed to create enough space for an implant crown with an anatomical design.

## Conclusions

It is concluded that under premise of preservation of sufficient bone to achieve primary stability of the implant, removal of the canines can be combined with immediate placement and provisionalization of the implant.

## Consent

Written informed consent was obtained from the patient for publication of this case report and any accompanying images. A copy of the written consent is available for review by the Editor-in-Chief of this journal.

## Abbreviations

CBCT: cone beam computed tomography
RP: regular platform
NP: narrow platform
